# Navigating local relevance in transdisciplinary research: Exploring climate and environmental change in the Tasiilaq region, East Greenland

**DOI:** 10.1007/s13280-025-02198-6

**Published:** 2025-07-16

**Authors:** Sophie Elixhauser, Jorrit van der Schot

**Affiliations:** 1 Department of Social and Cultural Anthropology, University of Vienna, 1010 Vienna, Vienna Austria; 2 Austrian Polar Research Institute (APRI), Vienna, Austria; 3 Department of Geography and Regional Science, University of Graz, Heinrichstr. 36, 8010 Graz, Austria

**Keywords:** Climate and environmental change, Co-creation, Greenland, Participation, Relevance, Transdisciplinary research

## Abstract

Transdisciplinary research aims to produce knowledge relevant to scientists and non-academic stakeholders alike, a challenging task given the parties’ divergent epistemologies and the attendant time and resource constraints. In Tasiilaq, East Greenland, our group of climate scientists and anthropologists set out to study climate and environmental change and the impact on the local community of changes in precipitation from less snow to more rain. We describe how our project team tried to make the project relevant to our collaborators, and the tension that arose between scientific and local relevance, which proved difficult to resolve. Our experience also points to some fundamental frictions in transdisciplinary research and limitations of conventional project and funding schemes. We recommend that transdisciplinary projects should be co-created by all partners from the outset to ensure equal participation and to avoid the difficult, sometimes intractable task, of rebalancing scientific and local relevance at a later stage.

## Introduction

Transdisciplinarity, understood as the inclusion of different actors and disciplines in the production of knowledge, has become a key tool for researching environmental issues. There is now a broad consensus that research on climate and environmental change requires conversations across the natural sciences, social sciences and humanities. Transdisciplinary research also involves non-academic partners, such as local and Indigenous communities, and aims to be relevant to both academic and non-academic collaborators (Rigolot 2020; Wylie and Murillo 2023). This type of research places a strong emphasis on the production of societally relevant knowledge, which in turn can be divided into practically relevant and policy-relevant knowledge (Pohl and Hirsch Hadorn 2007). Societally relevant knowledge should contribute to solving lifeworld problems in situations when knowledge is uncertain and the concrete nature of a problem is disputed. Practically relevant knowledge, in turn, must address threats directly experienced by local actors and contribute to the creation of practical solutions (Schikowitz 2020). The literature cites a wide range of challenges facing those doing transdisciplinary research, with these stemming from differing types of knowledge, (post-)colonial structures and hierarchies between scientists and non-scientists. While there are solutions—plural epistemologies, mixed methods and working at eye level with local research partners—they are particularly time-consuming and difficult to put into practice (Klenk and Meehan 2015; Elixhauser et al. 2024).

In this article, we analyse a research project considering environmental changes in an Inuit community in East Greenland, a setting that allowed reflecting on the challenges of aligning different forms of relevance in transdisciplinary research. We point out some underlying tensions in transdisciplinary research at large as well as the limitations of research projects whose duration is fixed at a certain number of years. In the focal region, Tasiilaq, our group of climate scientists and social anthropologists set out to study climate and environmental changes and the impacts on the local Inuit community of changes in precipitation from less snow to more rain. The transdisciplinary Snow2Rain project aimed to collaborate closely with the community and to integrate the perspectives of snow climatology, social anthropology and the knowledge and experiences of the residents (Tasiilarmeer in East Greenlandic). This approach raised several questions about relevance for the different partners, especially about the “local” relevance of our project theme.

We define local relevance as the relevance of our research, that is, the topics, research questions, methods, overall approach and results, to the people living in Tasiilaq, recognising that what is relevant may vary significantly for different groups of people within the community. Our “local relevance” corresponds largely, but not exclusively, to “practical relevance” as described above.

This article aims to (1) show how we, together with Tasiilarmeer, navigated the issue of "local relevance" throughout our project, (2) reflect on the challenges, potentials and limitations of this process and (3) more generally, discuss the tensions between different types of relevance in transdisciplinary research. We begin by introducing the theoretical framework, the study region and our methods. After a brief outline of the project findings, we give a detailed account of how we approached the issue of local relevance. We then go on to discuss our experiences within ongoing debates on transdisciplinarity and co-creation and conclude with some recommendations.

## Theoretical framework

The importance of localised forms of knowledge for environmental research has been emphasised for a long time (e.g. Danielsen et al. 2014; Schiøtt et al. 2021). However, despite increasing efforts, this knowledge—sometimes called traditional, ecological, local or Indigenous knowledge—is still not sufficiently recognised, accepted and integrated into research and decision-making processes (Yua et al. 2022). As Danielsen et al. (2014: 83) assert, in Greenland, as in other parts of the Arctic, “discrepancies between the authorities’ perceptions of the status of the environment and the local peoples’ knowledge and perceptions have, in some areas, led to frustration among community members and to limited local understanding and acceptance of government decisions.” Although some changes are taking place, it is still predominantly external researchers and institutions that carry out the research on Indigenous peoples’ lands, in the Arctic and beyond. This work is guided by research policies and funding from nation states or international bodies, which reproduce “relations of exclusion and domination established during the era of colonisation” (Herrmann et al. 2023: 19). However, in order to successfully bring together different knowledge systems, the research community at large must be willing to adapt and change existing processes, procedures and policies (Yua et al. 2022).

Seemingly in response to this call, one increasingly encounters more profound efforts to co-create research—based on a co-production of knowledge—that involve non-academic research partners at all the different stages of a project (Herrmann et al. 2023; Karlsdóttir et al. 2023). This appeal to collaborate at eye level is part of a larger trend in academia to make science more democratic and practically relevant (Gonzalo-Turpin et al. 2008). Indeed, various collaborative approaches have emerged, such as participatory action research (Hervé et al. 2023), community-based participatory research (Saxinger et al. 2018), citizen science (Tengö et al. 2021) or integration and implementation sciences (Bammer 2017). In the social sciences, anthropology in particular, giving research partners credit for their (active) role in academic knowledge production builds on decades of ethical and methodological discussion (Caplan 2003). In many natural science disciplines, collaborative approaches are rather new. Collaborative research explicitly emphasises the moral and practical commitment to achieve relevance “in a way that is responsive to the obligations that stakeholders pose to us, the researchers” (Klenk and Meehan 2015). Nevertheless, Wylie and Murillo (2023) argue that to date knowledge co-production has often not worked satisfactorily for non-academic stakeholders, with researchers often claiming that projects are co-created without really knowing what that means. For example, projects may be described as co-created if this applies solely to the implementation of the project, but not to the dissemination of the results or the development of the research topic. This problem is captured by Yua et al. (2022), who note that a key problem is confusing “the parts with the whole,” as truly co-produced knowledge and co-created research must encompass all the stages of a research project. Wylie and Murillo (2023) argue that such false claims are particularly harmful to Arctic communities, whose knowledge practices scientists have long marginalised and exploited. In Greenland’s national research strategy, the Government of Greenland (2022) underscores that research should first and foremost benefit Greenland. In this light, it is highly relevant to collectively address what knowledge co-production, collaboration and relevance mean for all partners involved in transdisciplinary projects.

Engaging non-certified experts in research, as Klenk and Meehan (2017) argue, is thus “fundamentally about matters of *relevance*”. Similar to our use of the term “relevance”, Cash et al. (2003) speak about *salience* to denote the relevance of information for stakeholders’ decision-making or choices affecting them. In considering different types of relevance, many scholars build on the distinction between scientific and societal relevance, as detailed above (Rigolot 2020). Relevance is not fixed in advance but has to be regarded as an outcome of the encounters throughout the research process (Klenk and Meehan 2017). Studies exemplifying the processes by which transdisciplinary research teams have integrated different types of relevance are rare (but see Gonzalo-Turpin et al. 2008; Schikowitz 2020). Such studies are important in investigating “why claims and aims for alternative ways of knowledge production often fail to work out in practice”, Schikowitz (2020: 221) argues. Answering this question has provided the impetus for this article, which builds on discussions on the decolonisation of research practices, currently the subject of much debate in the social sciences and increasingly finding its way into the natural sciences. In a case study from Greenland, we explore in detail the challenges, potentials and pitfalls of trying to bring together different types of relevance in transdisciplinary research.

## The Tasiilaq region

**Fig. 1:**
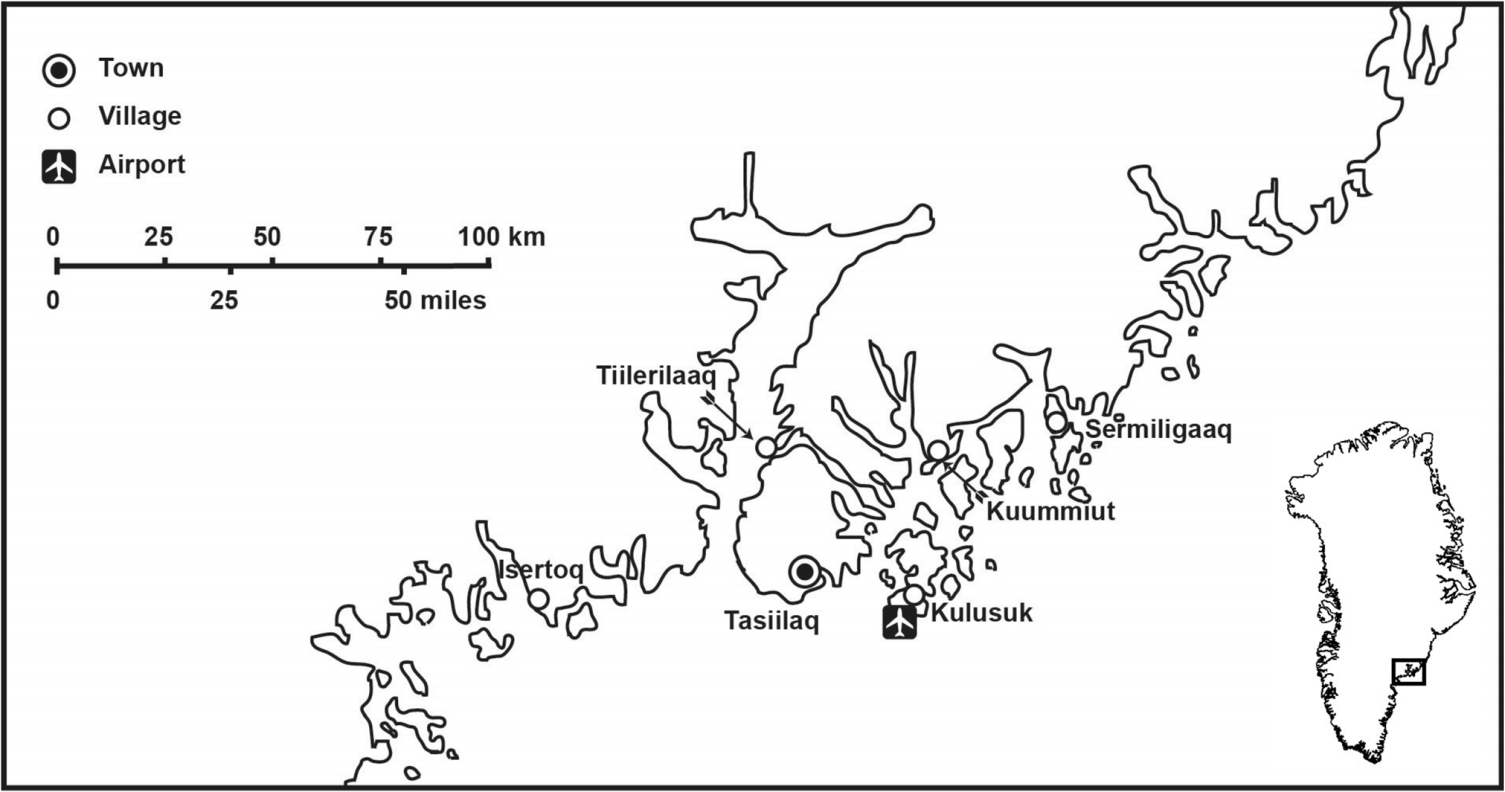
The Tasiilaq region in East Greenland (Map by Elixhauser)

The Tasiilaq region (Fig. 1) is one of two inhabited areas in East Greenland; as of 2025 it had 2546 inhabitants (Statistics Greenland 2025). The mountainous fjord landscape receives high amounts of precipitation and many storms: The *pilerngar*^1^ is a cold, hurricane-strength wind from the Greenland Ice Sheet; and *neqqajar* is a storm with northeasterly winds. The focus of Snow2Rain was the town of Tasiilaq (1758 inhabitants) (Statistics Greenland 2025—located on Ammassalik Island—and its surroundings. The island is also the location of a polar research station, Sermilik Station, a base for scientific research on the nearby glacier. It has recently been expanded and is now run jointly by Austria and Denmark.

For a long time, East Greenland was shielded from much contact with the outside world by the Arctic Ocean. The region was colonised but not until 1884, much later than in the case of West Greenland, where colonisation started in 1721. Hence, East Greenland’s social and economic development from a hunting society to a society mainly based on wage labour has taken place in a comparably shorter time. Today, the inhabitants live from fishing, tourism, handicrafts and the jobs offered by the Greenlandic Government, but there is also a high unemployment rate. Hunting is done mainly for subsistence. Most of the population of Tasiilaq would consider themselves *Iivit* (Inuit). Five to ten per cent of the population in Tasiilaq are Danes (and other foreign) residents or Greenlanders from other regions. East Greenlanders speak Tunumiusut, a language which lacks an official orthography and is unwritten. All children learn Kalaallisut (West Greenlandic, the country’s official language) at school as well as Danish and some English (Elixhauser 2018).

After being a Danish colony for several centuries, Greenland was officially "decolonised" in 1953 to become a self-governing territory of the Kingdom of Denmark. Throughout the 1960s and 1970s, resettlement programmes and initiatives to "modernise" Greenland along Danish lines were carried out, leading to many social problems and much criticism today. The growing demand for greater autonomy led to the granting of Greenlandic Home Rule in 1979, which was extended to self-governance in 2009. There has been increasing political and public debate about full independence from Denmark, an ambition which is currently hampered by Greenland’s financial dependence on Denmark. Greenland’s social and political system closely follows the Danish model, and this copy-paste system has been criticised by what is a growing decolonisation movement. The Danish language continues to play an important role in the country, as do the many Danish professionals in high-level positions, a situation which implies unequal power relations and polarisation. Post-colonial Greenlandic identity is a highly debated topic (Gad 2017).

Unequal power relations are even more of an issue in East Greenland because there are significant economic and social differences between the country’s East Coast and the more populous West Coast. The East Greenlandic population has the lowest per capita income in the country, and a low level of education, with many residents feeling politically underrepresented and looked down upon by the (West) Greenlandic population. Polarisation is therefore not only an issue between Danes and Greenlanders, but also between East and West Greenlanders (Elixhauser 2023).

## Snow2Rain

Snow2Rain (2020–2023) was a transdisciplinary project combining the perspectives of snow climatologists, social anthropologists and residents of Tasiilaq. The community is affected by climatic changes in various ways (Heide-Jørgensen et al. 2023), but there has been little research focussing on the experiences of the population in this region of Greenland (but see Buijs 2010), with some existing studies on the group of fishermen and hunters (Flora et al. 2019, Laidre et al. 2018, and most recently, Rathcke et al. 2025). In one range of activities within Snow2Rain, we tried to fill this gap by exploring environmental changes and particularly the transition in precipitation from snowfall to rainfall by incorporating different disciplinary lenses and ways of knowing. The project did not include climate change in the title and though precipitation trends are closely associated with climate change, we mostly avoided the term during fieldwork. Our rationale here was that we wanted to find out what residents’ direct experiences were rather than what they had heard about climate change through educational institutions or the media. The project involved a PhD student in snow climatology (van der Schot), a PhD student in social anthropology (Burdenski) and a senior researcher in social anthropology (Elixhauser). Most of the project fieldwork was carried out by the two PhD students, although Burdenski left the project before its completion. Elixhauser became more involved in the project activities in the second part of the project. The team worked closely together with members of the community in an effort to make the research relevant to the local population, although this relevance was not clearly defined in advance.

## Materials and methods

The project was designed to be transdisciplinary, building from the outset on close collaboration between anthropology and climate science researchers and our research partners in Tasiilaq. We applied a mixed-method approach, combining qualitative social science with quantitative climate science. Throughout the project, the climate scientists and the anthropologists (especially the two PhD researchers) spent a great deal of time in familiarising themselves with and participating in the research methods of the other discipline. For example, the anthropology PhD student learned how to carry out different types of snow measurements, and the climate science PhD student, engaged in participant observation and informal conversations, spending much more time in the field than is usual in his discipline. The transdisciplinary part of the project took the form of a series of meetings and group discussions in different formats (e.g. youth and community workshops, an exhibition), with a particular emphasis on being culturally sensitive and accessible to our local research partners. These activities took place during three periods of joint fieldwork in Tasiilaq in three consecutive years, with either two or three of the project researchers participating. This paper is based on the two authors’ experiences. In addition, Elixhauser draws on her long-term ethnographic field research in the Tasiilaq region since the mid-2000s.

**Fig. 2:**
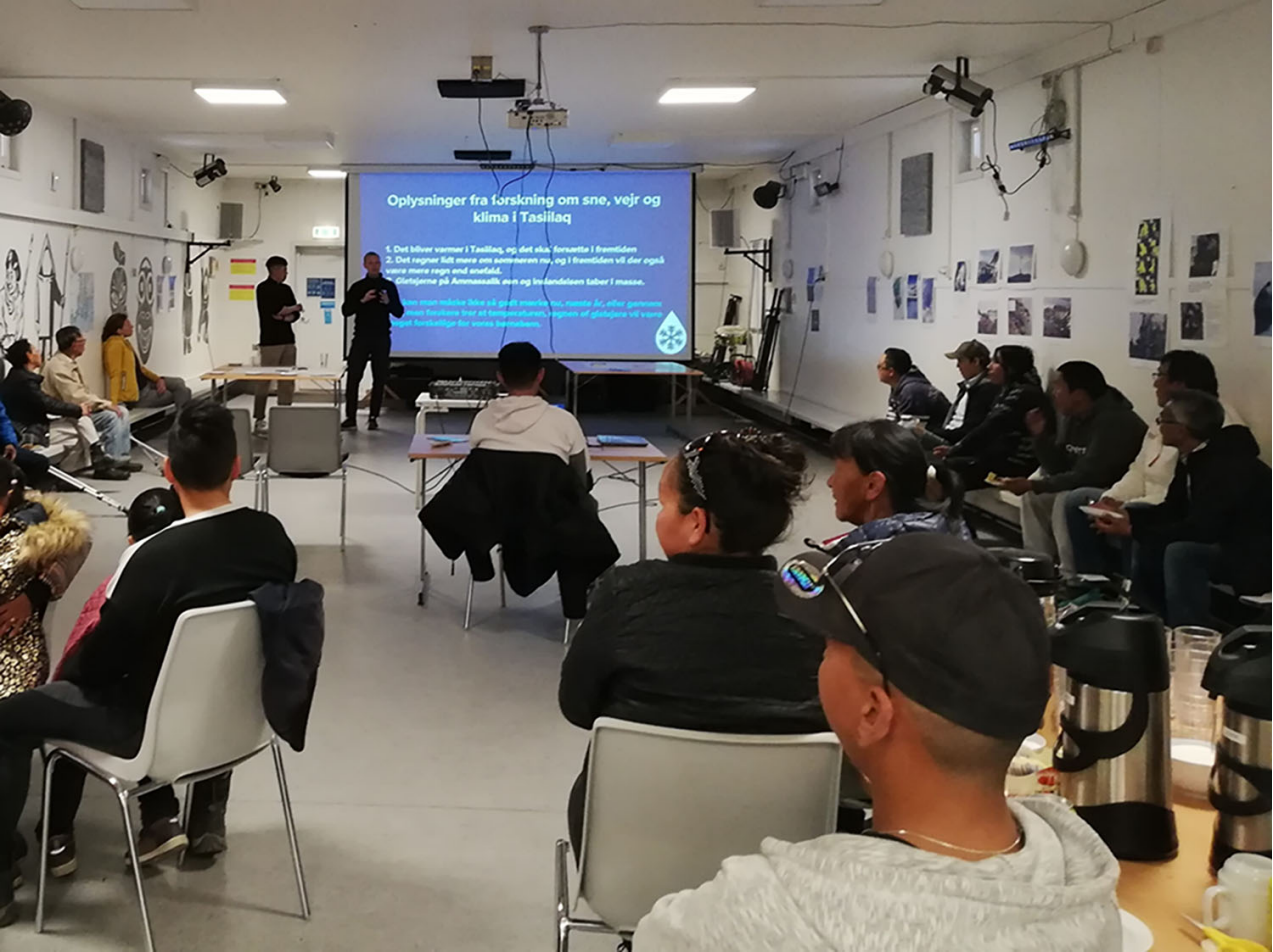
Snow2Rain workshop in Tasiilaq (June 2022) (Photo by Burdenski)

**Fig. 3:**
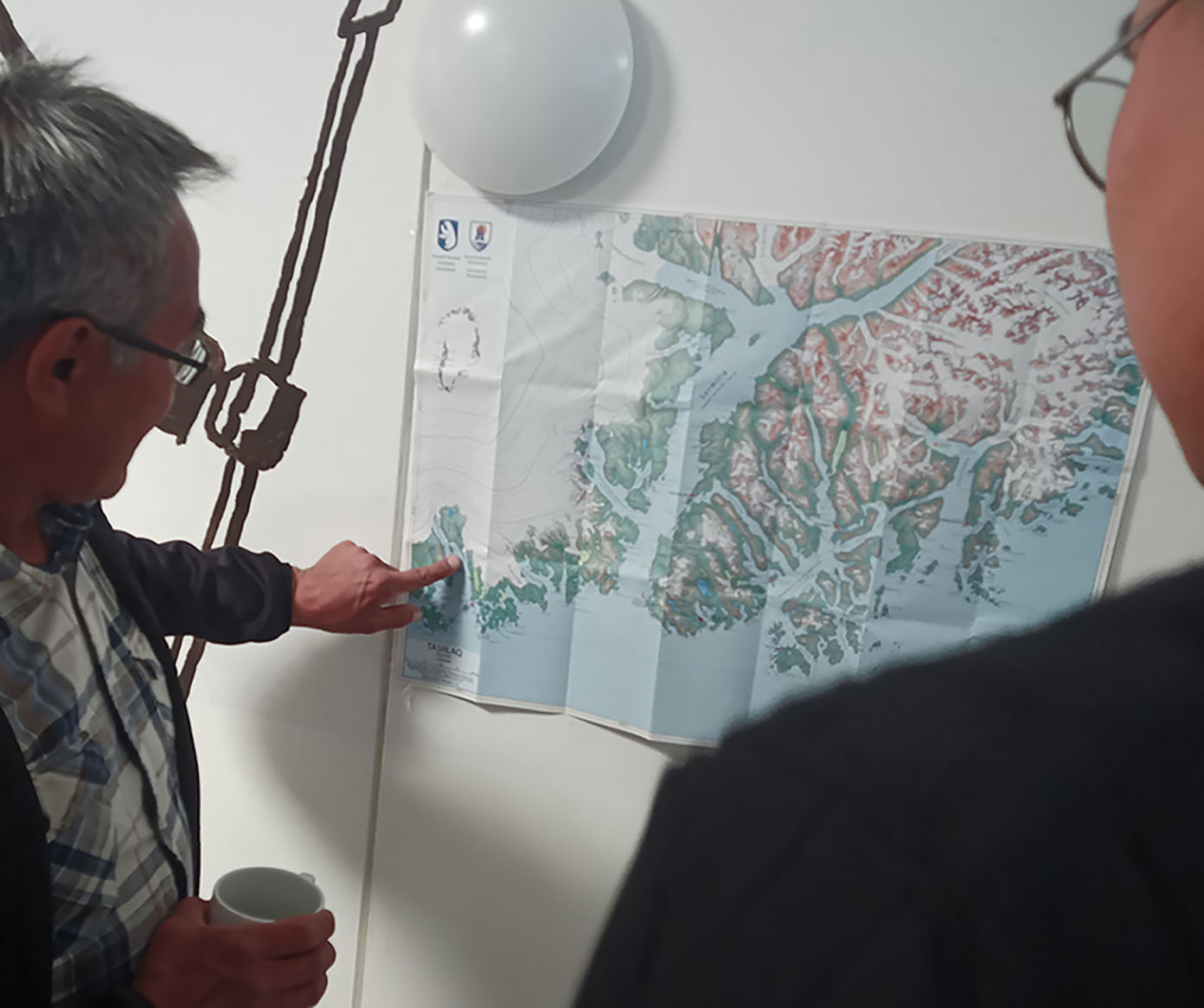
Tasiilaq resident sharing stories about environmental changes (June 2022) (Photo by Elixhauser)

The first one-month fieldwork period conducted by Burdenski and van der Schot started at the end of November 2021 and marked the start of a nine-month stretch of ethnographic fieldwork for Burdenski. A principal aim of the two researchers was to introduce themselves and their research in the community. They relied on participant observation, informal conversations and the observation of social and cultural practices. Furthermore, they had many meetings with local residents, organised a *kaffemik* (Greenlandic get-together), and closely collaborated with the local Ammassalik museum, which remained an important collaborator throughout the project. Another objective was to set up snow-measuring devices for the climate science side of the project. The snow measurements continued throughout the project and consisted of taking manual snow depth measurements, installing a snow sensor, and digging monthly snow pits. Throughout the project, climatological analyses were performed using datasets from the Danish Meteorological Institute’s Tasiilaq station (Cappelen et al. 2021) and outputs from the regional Arctic climate model RACMOv2.3 (Noël et al. 2019; see van der Schot et al. 2023). The second fieldwork period took place in June 2022, with all three researchers participating. They continued with participant observation, for example during the setting up of a community garden and the Greenlandic National Day celebration, with informal conversations and some semi-structured interviews, and with snow measurements. However, the main aim of this fieldwork was to organise a community workshop to discuss environmental change with local residents (Fig. 2). In addition, a workshop about climate change was held with young members of the community. The workshops were based on (climate) science communication as well as on interactive elements that facilitated a dialogue with the participants enabling the project researchers to learn from their experiences and specific interests (Fig. 3).

Elixhauser and van der Schot, the two authors, conducted the third field trip, which lasted for three weeks in April/May 2023. They set up a final exhibition in the Ammassalik Museum to share project findings and to spark further conversations with the community. The “Climate and Science” exhibition opened with a welcoming event and lasted for a week. Different groups of people were guided through the exhibition, such as two classes with teenage students.

### Snow, climate and environmental change in Tasiilaq

To contextualise the examples of how we navigated the theme of local relevance, we first give a brief summary of the project findings. The climatological analysis did not show any statistically significant trends in absolute precipitation in Tasiilaq (1958–2021). However, increases were found in the occurrence of rainfall compared to total precipitation in summer (1958–2015) and the altitude of the rain-snow boundary in the summer months (1958–2015) (van der Schot et al. 2023). The detected trends can be interpreted as rather subtle in terms of their climatological importance, which corresponds to residents’ perceptions. Based on many individual conversations and group discussions, we found that changing precipitation, from snowfall to more rainfall, was not an important topic among the residents of Tasiilaq. The fact that the winter of 2021–2022, during which the first field trip took place, had anomalous amounts of snow contributed to this. Many Tasiilarmeer were challenged to deal with the extreme snowfall and expressed their discontent. Referring to this winter, some residents reported doubts about a general trend towards less snow. Other individuals recounted that winter conditions nowadays have become more unstable, or have put the snowy winter in perspective, arguing that in former times there was always a lot of snow and not only in an exceptional winter like that of 2021–2022.

During the first two field trips, residents spoke little about the issue of rainfall, although some interlocutors mentioned that there is more rain in winter nowadays. This seeming lack of interest changed with our last field trip in April/May 2023, however. Shortly before our arrival, there was a continuous period of intense rainfall on top of snow and the increased runoff and snowy slush hampered access to various roads. The runway of Kulusuk airport was closed for 12 days. An increase in rain-on-snow events as part of global climate change has been reported from across the Arctic with various impacts on the environment and human livelihoods (Serreze et al. 2021). Citing an impact of this trend, one resident noted problems they encountered in reindeer hunting in the Kangerlussuaq area, West Greenland: “When there is rain on the snow and it freezes afterwards the reindeer cannot get through the ice layers to find plants to eat, and they are getting thinner” (pers. comm.). The heavy rainfall in the Tasiilaq region around this time in 2023 had other unforeseen consequences as well, such as the drowning of 14 sledge dogs on an ice flow close to the village of Kulusuk (Berthelsen 2023).

Of the climate change impacts investigated, temperature changes were more pronounced than precipitation trends. Station data as well as climate model output have revealed a significant increase in summer temperatures in Tasiilaq (van der Schot et al. 2023). This increase in temperatures has been experienced compellingly by residents of the region, and various residents shared salient experiences and memories. One man, for instance, recounted a boat trip he took with his wife the previous, hot summer, during which they were wearing no clothes at all (pers. comm.). In conversations about long-term temperature changes, some residents mentioned the large interannual variability. Our interlocutors noted myriad other environmental changes. Principal among these was that glaciers are retreating rapidly and the extent and duration of the sea ice is decreasing, which is of direct importance to East Greenlanders, who have to travel across the ice. The number of months when snow scooters and dog sledges can be used has decreased significantly. Among other impacts, this has led to a decline in the number of sledge dogs, an important element of Greenlanders’ collective cultural identity (Egevang 2020). Less ice cover on the fjords means greater reliance on boats, which poses risks to equipment and safety. Some residents also brought up possible advantages of climate change, for instance, routes that they would be able to sail when the glaciers are gone. Residents reported a variety of other observations, including changes in animal abundance, such as new whale species in the region, new environmental phenomena such as thunder, and an increase in the number of earthquakes.

Generally speaking, we repeatedly encountered doubts among our interlocutors about whether climatic changes are indeed human-caused. One participant in our youth workshop asked, “What about the ‘ice ages’, and the ‘naturally’ warmer and colder periods returning ‘every 100 years or so’” (pers. comm.)? For many participants, scenarios presented in one of our workshops showing how different the Greenland ice sheet might look in future depending on human activity were difficult to relate to; the role of human impact in the scenarios was particularly perplexing. A recent Greenland-wide survey on perceptions of climate change, based on the idea that climate change is caused by humans, speaks of a “gap between the scientific consensus and coastal Kalaallit [Greenlanders’] views of climate change” (Minor et al. 2023). Interestingly, this gap is more pronounced in Northwest and East Greenland, the two remotest regions of the country. Our findings accord with these results.

## Results

### From snow to rain: navigating local relevance

As early as the initial fieldwork in winter 2021, it proved difficult to connect with residents on the project theme, the transition from less snow to more rain in the past, present and future. Indeed, a Danish resident voiced doubts about the local relevance of our theme during the introductory *kaffemik*. She pointed to other climatic changes more relevant locally, for example the decreasing sea ice in the bay, which influences her and other residents’ snow scooter routes (pers. comm.). Many residents did not know what to expect from Snow2Rain, and it took time to introduce the project and to build trust. This reticence was also apparent when the project sought a local assistant to support the snow measurements, a procedure that proved difficult and took several months (Burdenski, pers. comm.). As white researchers from a Western country, we operate within a context marked by post-colonial power inequalities, which we believe is one reason it took a long time to build trust and find project assistants. During the second field trip, Burdenski had been in Tasiilaq for six months and was well integrated within the community and van der Schot returned. Now part of the team, Elixhauser could build on old relations in town, and we could sense positive changes in terms of trust and a positive attitude towards our project team. Yet, the difficulties in approaching the topic of precipitation changes persisted. One reason for this, we believe, might be the differences between people’s everyday experiences and the abstracted long-term trends relevant to climate science. As various anthropologists have pointed out, people perceive weather changes, but they do not directly perceive climate change (Strauss and Orlove 2004). Climate change is recorded and does not emerge from lived experience, whereas weather is experienced in a multisensory and embodied way. Experiences of weather (events) become woven into the narratives and personal life histories of the inhabitants of particular places (Ingold and Kurttila 2000). Accordingly, residents’ accounts of weather changes or unusual weather or environmental events were often linked to individual stories from their lives, and questions about long-term trends were more difficult for some residents to answer and were often linked back to similar events throughout their lives.

The difference between weather and climate came up several times in our workshops and discussions with Tasiilarmeer, and several interlocutors, especially younger people, could not explain it. While in climate science there is a clear distinction between the two categories, in Tunumiusut both terms are translated using the term *tsilar* (*sila* in Kalaallisut). *Tsilar* means “everything outside the house”, such as the weather, the air, the universe and the world, and it is also the word for “intelligence” and “reason”. To translate and differentiate “climate”, as our research partners explained, the word *pitsusaa* is added (*tsilar pitsusaa; silap pissusaa* in Kalaallisut), which is translated as “quality” or “behaviour” (Oqaasileriffik and the University of Chicago 2018). This Indigenous concept of *tsilar* suggests that categories of weather, climate and other environmental phenomena, seen as detached from the human world, are a Western intellectual construct. While it has been noted that the idea of climate as a category in its own right is quite new in Greenlandic society (Bjørst 2011), we do not want to argue that East Greenlanders do not distinguish long-term trends in weather from individual weather events. However, this distinction is expressed through people’s own cultural framework and an epistemology based mainly on local experience, which differs from science (Krupnik et al. 2010). Accordingly, we found that residents’ knowledge of the binary Western category of climate and weather varied greatly depending on their level of education and other individual factors.

Another factor explaining why our project topic seemingly lacked local relevance is that speculation about future change is not common among Tasiilarmeer. This tendency was observed in Snow2Rain and Elixhauser (2018) has argued for its existence elsewhere, referring to her previous ethnographic fieldwork in East Greenland. It has also been reported for other Inuit communities. For example, Bates (2007) reports from his fieldwork in the Canadian Arctic that many Inuit did not like to make predictions or plan far in advance, at least not in a linear way “with neat chronologies linking events in the past, present, and futures” (Bates 2007: 88). Although different scientific paradigms are becoming more important in environmental science (e.g. Preiser et al. 2018), linear causality continues to play a central role in Western thinking and science.

A key consideration at this juncture is that the Inuit and Tasiilarmeer live in the Arctic, which remains dangerous and unpredictable because of rapidly changing weather, environmental hazards and unpredictability, combined with recently introduced uncertainties about modern technologies. Bates explains that accepting that the future cannot be known, the Inuit in the Canadian community where he spent time (and, we would argue, people in East Greenland) are highly prepared for uncertainty and show an acute awareness of the future. They focus on “opening up a path in the present to the future, rather than becoming overly fixated on the future before it has arrived” (Bates 2007: 90). In climate science, however, projections about future changes are common and projected changes in precipitation were part of the motivation for developing the Snow2Rain project. While many participants were interested in the projections that we presented and asked questions about scientific explanations and findings, they largely reserved any thoughts they had about the future, behaviour which might be attributed to these different philosophies of time.

As our research topic proved somewhat unwieldy, we broadened the project theme to include issues that our local research partners deemed more relevant. Instead of focusing primarily on the trend from less snowfall to more rain, we chose to address environmental changes in the region at large.

### The example of earthquakes and our strategies for increasing local relevance

On 23 June 2022, we held a workshop in the community hall to share interim project results and learn about residents’ interests and knowledge. The participants raised a variety of topics, such as changes in the abundance of animals, sea and fjord ice, and weather/environmental phenomena. One topic that was mentioned several times was the increasing number of earthquakes experienced in Tasiilaq. One elderly resident was eager to know whether the earthquakes are connected to global climate change, and we promised to bring back relevant scientific information to the community on our final field trip. The literature studies on earthquakes in the region showed that the number of earthquakes in Greenland is indeed increasing and that this trend is (mostly) related to glaciers (Ekström et al. 2006). Specifically, Ekström et al. (2006) describe a new type of earthquake, a glacial earthquake, connected to calving. The glacial earthquakes that are felt in Tasiilaq likely come from the nearby Hellheim glacier, and occur because the glacier has retreated close to its grounding line, where the ice detaches from the underlying bed. If this is the case, the increase in glacial earthquakes is linked to climate change but not linearly.

In our final “Climate and Research” exhibition, we incorporated this information alongside other themes that proved to be of particular interest to residents. A few days before the opening of the exhibition, a large earthquake (4.5 on the Richter scale) occurred in Tasiilaq, and we integrated some data about it in the exhibition at the last minute, which sparked considerable interest. Similarly, several times throughout our fieldwork, people approached us asking about the Sermilik Station, as new construction and changes had been taking place there after Austria became a co-operator. In response to this interest, we integrated information about the station into the community workshop and the exhibition. In yet another case, several residents reported that they had experienced changes in the occurrence of the storms *pilerngar* and *neqqajar*, with more and stronger *neqqajar* taking place today, and showed an interest in a scientific explanation. Here, we responded by including a research poster in our exhibition with the relevant scientific findings. We view the integration of these new topics into the scope of our research project as adjustments that led to increased local relevance. It was residents who brought up the key issues, and our inputs were enthusiastically discussed by and with residents who participated in our project activities. Generally speaking, research in and about East Greenland has often been poorly communicated in the past, and proper communication is therefore important to the community council, as the Tasiilaq district manager, Hjørdis Viborg, mentioned to Elixhauser (pers. comm.). We heard similar experiences from other community members who were pleased by our efforts in communication and collaboration. This feedback from the community prompted us to include in our exhibition a map with information about past research activities from various disciplines.

### Limitations on creating local relevance

Above we have highlighted some modifications to the project that may well have increased its relevance to the residents of Tasiilaq. Yet, this is not to say we did not encounter numerous challenges and limitations. Our strategy of broadening the project’s scope by integrating issues deemed more relevant by Tasiilaq residents was not feasible for all aspects of our methodology. For example, the climatological component had to remain essentially as initially planned, in contrast to the anthropological and interdisciplinary parts, which could be adapted more easily. Anthropology follows an inductive approach, which means that research topics evolve and may change in the course of a researcher’s fieldwork. In climate science, which mostly follows a deductive approach, such a procedure is not common or readily feasible. In our project, climatological work relating to the “new” topics mentioned above was always an addition to the activities described in the work packages. These were planned, prepared and started at the very beginning of the project, formed a core part of van der Schot’s PhD and reports on their progress had to be submitted to the funding agency. Climatological field measurements in a remote Arctic environment require extensive planning and, in our case, necessitated sending snow-measuring equipment by ship from Austria to East Greenland, work that has to be organised well in advance. As we only learnt what would be more relevant themes in the second half of the project cycle, the project had already advanced too far to undertake new climatological measurements. Even the analysis of existing datasets is often labour-intensive and difficult to carry out in addition to other research activities. Accordingly, we were able to include some minor, new climatological analyses (e.g. wind speed data for our exhibition), but it was difficult to go into depth. Hence, our case exemplifies the potential impact of differences in the methodology of scientific disciplines involved in transdisciplinary research, where some rely on an inductive approach while others adopt a deductive approach. More general constraints are the conventional project structure and timeline. Some of the time challenges can be overcome if a project is part of a long-term collaboration with local stakeholders that lasts longer than a research cycle, that is, a transdisciplinary research programme or unit where lessons learned from previous projects (covering different disciplines) can be applied to the next, even if (some) researchers may change.

## Discussion

We encountered various challenges due to the different epistemologies that attend the transdisciplinary process, one dilemma being the need to integrate the different temporalities of residents’ narratives of weather events and climatological long-term trends. For some of our interlocutors, it proved difficult to bring these time scales together, for instance when singular events such as the cold winter of 2021–2022 seemed to contradict the long-term global warming trend. Residents who were more familiar with the scientific contexts were able to readily switch between the different perspectives. It is important to add that the distinction between these time scales and epistemologies is far from clear-cut, and, as Hastrup (2013: 149) wrote in her analysis of scales of attention in ethnographic fieldwork in the Arctic, “diverse scales may become part of the same picture, or narrative”.

Many researchers have encountered a "discrepancy" or "disconnect" between scientific knowledge and local knowledge systems (e.g. Baztan et al. 2017; Danielsen et al. 2014). Such a discrepancy was evident when we contrasted weather and climate in our workshops, a juxtaposition with which some residents were unfamiliar. As mentioned above, in Tunumiusut, *tsilar* is a term used to talk about weather and climate, but the concept subsumes some very human characteristics as well. It thus differs in its basic premises from the nature/culture divide that forms the basis for Western thought and (climate) science (Bjørst 2011). Verran (2010) has pointed out that epistemological differences can form the basis of effective collaboration when they are collaboratively explored in an early phase of a project. However, given the time that it took the two PhD researchers to start their collaboration with Tasiilarmeer, following Verran’s suggestion was not feasible: collaboratively exploring how *tsilar* and other East Greenlandic terms referring to weather, climate and environmental phenomena could be brought together with climate science would certainly have gone beyond the scope of our initial project phase.

Exploring synergies between different epistemologies has a direct impact on the question of relevance. The local relevance of our research encompassed various elements of societal relevance as well as the practical issues that had an immediate impact on people’s everyday lives; hence, practical relevance featured centre-stage. For example, during the winter of 2021, extreme snowfall was a topic of discussion for many residents, as the heavy snowpack meant people had to remove snow from their roofs and doorsteps daily. During the spring of 2023, heavy rain events caused various problems in the region, such as blocked roads or the drowning of sledge dogs. These experiences influenced the relevance that the residents saw in our project topic, for themselves and their region. However, whereas the snowy winter of 2021/2022 led some residents to doubt the relevance of our research theme as it did not seem to support the thesis of a precipitation trend towards less snow, the heavy rain event of 2023 confirmed the relevance of our project for some. These examples can clearly be placed within the category of practical relevance. Some residents also thought in broader terms, mentioning issues that did not derive from their immediate everyday experiences. Examples include a resident’s question about plastic in the ocean (which this person had heard about in the media but not experienced personally) or another’s asking about ozone holes and how this phenomenon relates to precipitation and climate change. Here it is somewhat more difficult to determine the nature of the relevance in the questions, as some of the questions showed scientific curiosity and some perhaps an underlying interest in possible changes in environmental policies affecting East Greenland. In general, residents did not talk explicitly about policy issues in our workshops and interviews, and therefore policy relevance could not be captured. For example, residents repeatedly mentioned changes in animal abundance, including the fact that certain whale and seal species had moved further north, an issue directly linked to the much-debated quota system that governs the hunting of marine mammals in Greenland. This link, however, was not discussed in the frame of our project activities.

Overall, the Snow2Rain project revealed that the extent to which the residents of Tasiilaq feel affected by a long-term shift in precipitation towards more rain is limited. This explains some aspects of the challenges we experienced in navigating local relevance. For Tasiilarmeer, the subtle, long-term precipitation shift is not perceived as a major threat, although recurrence of singular, extreme rainfall events, such as that in spring 2023, would pose risks to certain parts of the community. On balance, adopting a focus on case studies and extreme events might have helped to make the climatological perspective more relevant to Tasiilarmeer. Among other things, this approach would be in line with McDonald et al. (2015) recommendation to reduce the psychological distance of climate change.

Different types of relevance, as we have shown, may not always align with each other. Since we only learnt about the topics deemed more relevant by Tasiilarmeer during fieldwork, a significant tension arose between the project’s scientific and local relevance. This was particularly challenging for the climate scientists in the project, who had to cope with trade-offs due to responsibilities towards different actors and interests: On the one hand, they felt obliged to carry out the tasks pre-identified in the work packages, such as producing scientific articles answering the original research questions, based on measurements that were started in an early project phase; on the other hand, they were expected to carry out locally relevant research in close collaboration with partners from Tasiilaq. Among the factors triggering the tensions between scientific and local relevance was the fact that our research framework had been finalised before the concrete project-related collaboration with Tasiilarmeer began and that it was not possible to greatly modify the climatological parts later on. Our experiences also lend credence to Schikowitz’s (2020) argument that the tensions between different types of relevance in transdisciplinary research are exacerbated when collaboration goes beyond one’s own discipline.

Hence, major limiting factors in our attempt to align scientific and local relevance were the challenges of adapting research questions throughout the project, which required integrating two different academic disciplines, and more generally, the need for more time to accommodate collaboration with and equal participation of the non-academic stakeholders. Various authors point out that a key challenge of transdisciplinary research is its time-consuming nature, both in terms of collaborating across disciplines and with non-academic partners, such as Indigenous communities; academic structures and funding agencies do not usually provide for sufficient time (Saxinger et al. 2018; Steger et al. 2021). Our experience supports this argument, and we therefore strongly support Doering et al.’ call for “better-focused and long-term funding structures and mechanisms that can facilitate the development of relational integration for Indigenous rights holders and non-Indigenous research partners” (2022: 4).

Various publications about inter- and transdisciplinary research in the Arctic have highlighted the potential benefits of research that from the very outset is co-created with local partners, an approach that would encompass a stage of deciding upon a topic (Degai et al. 2022; Herrmann et al. 2023). Gonzalo-Turpin et al. (2008) argue that to achieve practical relevance a diversity of stakeholders should be involved in problem definition and problem-solving. Co-creation in transdisciplinary research is closely related to the principle known as “two-eyed seeing” (Reid et al. 2021), which highlights the importance of giving equal consideration to multiple ways of knowing. This approach, we argue, can be of major benefit in successfully meeting some of the challenges we experienced. These included the difficulties the two PhD researchers had in introducing the project to the community and the time it took them to overcome initial scepticism and build good relationships—both in their first project in East Greenland. While we discovered ways to adapt our research topics in the course of the collaborative project activities, we could not make these changes, because it was already too late in the project cycle. We thus argue that to align different types of relevance, transdisciplinary projects need to engage with communities around the specific research topic at a much earlier stage. Co-creating a research topic that is (to some extent) relevant to all actors involved and building trust in the research approach should be a prerequisite for, not an outcome of research.

If we are to gain insights into the co-creation of research and transdisciplinarity, we must revisit the definition of transdisciplinary research. Despite decades of discussion, there is currently no consensus about an exact definition. Widely shared core elements are that transdisciplinary research focuses on a societal problem, is based on interdisciplinarity and involves some form of collaboration with non-academic stakeholders (Jahn et al. 2012; Schikowitz 2020). Somewhat surprisingly, collaboration in transdisciplinary research is understood in various ways. Some argue that engagement with society does not necessarily have to be in-depth; they see it as consulting societal actors; Others, including us, speak of participation on an equal footing (Mobjörk 2010). The second orientation is based on recognising non-academic stakeholders’ knowledge as equally valid to scientific knowledge and the argument that transdisciplinary research always includes a process of mutual learning (Jahn et al. 2012). We do not want to categorically rule out a consulting approach, as it might make sense in some contexts if agreed upon by all participants, for instance when academics work in their own country or cultural context. When collaborating with Indigenous communities, however, who have long suffered (and still suffer) from marginalisation, power inequalities and the exploitation of their knowledge, we think that it is of utmost importance to follow a “participatory” transdisciplinarity (Mobjörk 2010). This participatory transdisciplinarity should be based on the principles of co-creating research with all academic and non-academic stakeholders, in a process that must encompass all of the stages of a project and a joint definition of the problem at the very outset.

The challenges we have cited also point out some general limitations of the increasing "projectification" of university research (Fowler et al. 2015). The common project structure is based on regulated and controlled, linear time schemes, which, as Dollinger (2020: 675) writes, “may hinder more creative or open problem-solving techniques.” Reminding us that time is always heterogeneously experienced, she speaks of alternative timescapes to projectification, juxtaposing "project time" and "process time". The latter is not based on linear progress and “may or may not build on existing work, may be subject to periods of inactivity, and may change course as new ideas come forth” (Dollinger 2020: 676). Yet, as our experiences have shown, it may be challenging to integrate all the processes necessary and their specific temporal requirements—the process time—within the frame of a project. This argument is most crucial when considering the differing timescapes of non-academic partners involved in transdisciplinary research, in our case the Tasiilarmeer, who did not think in this three-year research project “box” when interacting with us. Accordingly, finding common ground and building trust among the academic and non-academic research partners did not take place along the lines of our project timescape.

In their analysis of project management schemes in university research, Fowler et al. (2015) detect a separation between research praxis and its presentation, for example that directed to funding bodies. This leads to “front-stage” and a “back-stage” communication in university research, following the sociologist Goffman’s (1959) famous distinction, where front-stage behaviour occurs when others are watching, and back-stage behaviour when no one is watching. The latter form of communication is based on “a mutual understanding of the discrepancy between how research is planned and presented, and how it is actually conducted” (Fowler et al. 2015: 28). This also applies to our research project. Fowler et al. (2015: 31) conclude that “a projectification of university research should arguably call for tools that acknowledge the processual nature of venturing into the unknowns, tools adjusted to a process whereby directions as well as deliverables are under constant reformulation, and the guiding questions as well as the contributions may often only be formulated retroactively”. While we fully support this call, which speaks directly to what we wrote above about the need for a pre-project phase, our experiences also show that such an approach cannot be implemented equally well in all disciplines.

## Conclusion

We have examined in detail the process of collaboration between different disciplines and between scientists and local stakeholders in our project on climate and environmental change in East Greenland and analysed our adaptations of the research theme along the way. In the process, we have identified several of the challenges and tensions known to constrain transdisciplinary research. Time constraints, epistemological differences, capacity issues and unequal power relations are a few examples. The main finding of this article is that these challenges and tensions play a key role in the difficulties of reconciling different types of relevance in transdisciplinary research—this reconciliation being a major strength claimed by this collaborative form of research. In order to reconcile scientific relevance and the relevance of local or non-academic stakeholders—here called “local relevance”—a common definition of the research problem is of paramount importance. If collaboration starts too late, that is, after the project theme has been defined and the project cycle has started, it may indeed be too late for the adaptations needed to make the transdisciplinary project a truly participatory and co-created one; this was our experience in the focal project. In short, the joint development of a common definition of the research problem, topic and questions must take place before a project starts. Yet, this essential pre-project phase is hardly taken into account in the conventional project cycle and funding structures.

Our project is a case in point. It was only after it had been approved by the funding agency and the academic team had been formed that our project-specific collaboration with Tasiilarmeer could begin. After the first project activities in Tasiilaq—which were even more delayed by COVID-19—we learnt that changing precipitation was not a particularly important issue for the community, even though residents had been dealing with extreme snowfall and extreme rainfall. The project’s climatological data support this point, although more significant changes are projected for the future. Temperature changes in Tasiilaq were found to be more pronounced than changes in precipitation trends, and here both climatological measurements and Tasiilarmeer’s experiences accorded closely with one another. In general, participants reported a variety of environmental changes in the Tasiilaq area, most of which were found to be more locally relevant than changes in precipitation. Because of the limited local relevance of the original focus of our transdisciplinary project, we began to broaden the scope of our research to include issues that the residents considered more relevant. These included the increasing number of earthquakes, changing storm patterns and developments at the Sermilik Station. At the same time, these collaborative activities gave rise to challenges stemming from the different temporalities and epistemologies relevant to Tasiilarmeer, anthropology and climate science, for example in terms of conceptualising long-term trends or talking about the future. This created a tension between scientific and local relevance that was difficult to resolve within the limited time frame of our project, especially for those working on climatology. This tension between local and scientific relevance meant that locally relevant research had to be carried out in close collaboration with Tasiilarmeer, while at the same time, the original climatological research questions had to be answered to fulfil the PhD and funding requirements.

These experiences have led us to argue that a key factor in successfully reconciling scientific and local relevance in transdisciplinary projects is the involvement of non-academic partners in all phases of a project, including the design phase. The early involvement of non-academic partners, which is part of the current call for co-creating research, and co-producing knowledge, helps to balance unequal power relations and to make clear in advance the willingness and capacity partners have to collaborate in the project. The approach of co-creating research together with non-academic partners currently has a largely unrealised potential to empower local and Indigenous communities to shape their research agenda, with positive implications for trust and collaboration, as has been pointed out elsewhere. The co-creation process addresses some of the challenges experienced from the outset and we argue, removes the need for adjustments to realign scientific and local relevance at a later stage. At best, adjustments made at a later stage may add a modicum of local relevance, but they cannot retrospectively change the design, set-up or theme of a transdisciplinary project. When working with Indigenous communities, transdisciplinarity based on the equal participation of non-academic partners in all stages of a project is crucial to establish democratic procedures and ensure that the research effort in no way perpetuates post-colonial structures and power inequalities.

## Data Availability

We do not analyse or generate any datasets, because our work is based on our experience rather than quantitative data from questionnaires or interviews.

## Footnotes

1 We use East Greenlandic ways of writing throughout this article. This mode of writing developed and continues to develop in East Greenlanders’ informal channels of communication.
