# Publisher Correction: Navigating local relevance in transdisciplinary research: Exploring climate and environmental change in the Tasiilaq region, East Greenland

**DOI:** 10.1007/s13280-025-02242-5

**Published:** 2025-08-30

**Authors:** Sophie Elixhauser, Jorrit van der Schot

**Affiliations:** 1 Department of Social and Cultural Anthropology, University of Vienna, 1010 Vienna, Vienna Austria; 2 Austrian Polar Research Institute (APRI), Vienna, Austria; 3 Department of Geography and Regional Science, University of Graz, Heinrichstr. 36, 8010 Graz, Austria

**Correction to: Ambio** 10.1007/s13280-025-02198-6

The production team missed to delete “...(anonymised)” in the sentence “The focus of …(anonymised) Snow2Rain was the town of Tasiilaq (1758 inhabitants) (Statistics Greenland 2025—located on Ammassalik Island—and its surroundings.” appearing under the section “The Tasiilaq Region”.

The original article has been corrected.

